# Regulation of neuronal development and function by ROS

**DOI:** 10.1002/1873-3468.12972

**Published:** 2018-01-26

**Authors:** Matthew C. W. Oswald, Nathan Garnham, Sean T. Sweeney, Matthias Landgraf

**Affiliations:** ^1^ Department of Zoology University of Cambridge UK; ^2^ Department of Biology University of York Heslington York UK

**Keywords:** axon, cytoskeleton, dendrite, NADPH oxidase, nervous system, neuronal polarity, pathfinding, plasticity, reactive oxygen species, synapse

## Abstract

Reactive oxygen species (ROS) have long been studied as destructive agents in the context of nervous system ageing, disease and degeneration. Their roles as signalling molecules under normal physiological conditions is less well understood. Recent studies have provided ample evidence of ROS‐regulating neuronal development and function, from the establishment of neuronal polarity to growth cone pathfinding; from the regulation of connectivity and synaptic transmission to the tuning of neuronal networks. Appreciation of the varied processes that are subject to regulation by ROS might help us understand how changes in ROS metabolism and buffering could progressively impact on neuronal networks with age and disease.

## Abbreviations

BH_4_, tetrahydrobiopterin

CamKII, calcium/calmodulin‐dependent kinase II

CRMP2, collapsin response mediator protein 2

ER, endoplasmic reticulum

ERK, extracellular signal–regulated kinase

Grx1, glutaredoxin 1

HFS, high‐frequency stimulation

IP3Rs, inositol‐3‐phosphate receptors

JNK, Jun‐N‐terminal Kinase

LTD, long‐term depression

LTP, long‐term potentiation

MICAL, molecule interacting with CasL

MsrB, methionine sulfoxide reductase

NGF, nerve growth factor

PKC, protein kinase C

PP, protein phosphatase

PTEN, phosphatase and tensin homolog

PVIs, parvalbumin‐expressing inhibitory interneurons

redox, reduction–oxidation

ROS, reactive oxygen species

RyRs, ryanodine receptors

The increase in atmospheric oxygenation is presumed to have set the pace of evolutionary change. The symbiotic acquisition of mitochondria 1.45 billion years ago generating the eukaryota further allowed diversification *via* the efficient metabolic use of diatomic oxygen. Reactive oxygen species (ROS), highly reactive molecules and free radicals derived from molecular oxygen are produced as natural by‐products of normal respiratory metabolism, with the major source being the mitochondria. Mitochondria leak bursts of ROS as a function of respiration 1 pointing to a link between metabolism and ageing‐related damage. Other subcellular locations of ROS production continue to be identified and include the endoplasmic reticulum (ER) 2, peroxisome 3, the cytosol 4, plasma membrane 5 and extracellular space 6. However, there is a growing opinion and body of evidence that ROS can act as physiological signalling molecules. In this review we will focus on the role of ROS as a physiological signal in the nervous system during development and as a regulator of neuronal function.

By their highly reactive nature, ROS are damaging to the cell, oxidising proteins, lipids and DNA and are normally regarded as detrimental to cell function. Indeed, an overwhelming of the defences against ROS is termed oxidative stress and is commonly associated with cellular damage seen in neurodegenerative disorders, including Parkinson's 7 and Alzheimer's disease 8. ROS and the accumulation of ROS‐related damage are also associated with ageing; oxidised lipids, DNA damage and the accumulation of lipofuscin (the ‘aging pigment’, autofluorescent material found in endosomes consisting of oxidised lipids, proteins, transition metals and senescent mitochondria) 9. Increasing evolutionary complexity and concomitant demand for oxygen‐dependent energy production *via* reduction–oxidation (redox) reactions also produced a diversification in defence mechanisms against ROS. This is particularly notable in energy demanding tissues such as heart and liver. The nervous system is anomalous in this framework as nerve cells are very energy demanding yet at the same time inadequately equipped with antioxidant defence 10, 11. Interestingly, much of the ROS defence within the nervous system occurs in glia 12. It is becoming increasingly apparent, however, that evolution may have made a virtue out of a necessity and co‐opted ROS for cellular signalling mechanisms. For such a framework lowered antioxidant defence or ROS buffering in neurons would be permissive and necessary.

## Regulation of ROS in the nervous system, the case for the defence

Studies of ROS in the nervous system have primarily focused on ROS as damaging agents and the defence against ROS. Protection against ROS is mediated by multilayered constitutive and adaptive forms of defence. In the brain, static defences against ROS generally are seen to be (a) constitutive and enzymatic as seen in the standing high concentrations of enzymes such as superoxide dismutases, catalases, thioredoxin reductases and glutathione peroxidases or (b) constitutive and nonenzymatic as mediated by defence molecules (termed ROS scavengers) present in the cell, *via* synthesis or diet, such as alpha‐tocopherol (vitamin E), ascorbic acid, ß‐carotene and tetrahydrobiopterin (BH_4_). BH_4_ is a molecule of particular interest. For example, BH_4_ is a highly sensitive scavenger of H_2_O_2_ and Hydroxyl ions, while also important for the synthesis of the neurotransmitters dopamine, serotonin and noradrenaline. This suggests a functional link between ROS abundance, buffered by BH_4_ levels, and neurotransmitter function (for review see 13). A second set of defence mechanisms is adaptive and mediated by transcription factors 14. Two pathways are prominent: (a) the NRF2/Keap system, predominant in glial cells 12 and (b) the Jun‐N‐terminal Kinase (JNK)/AP‐1 system, seen to be a major protective mechanism in neurons. Both promote the transcription of genes encoding antioxidant response proteins. For example, NRF2 promotes the expression of glutathione‐*S*‐transferases in glia 15 while in neurons AP‐1 activation upregulates sulfiredoxin 16. NRF2 activity in glia mediates neuronal protection in a nonautonomous manner, partly through the ensheathing nature of symbiotic glial–neuron interactions. Indeed, keeping NRF2 function low in neurons allows dendritic and synaptic development and their regulation *via* redox‐sensitive signalling pathways, such as JNK/AP‐1 and WNT 17.

### Neuronal polarity

Most neuronal cell types are explicitly polarised, endowed with a major axonal neurite that mediates long‐range connectivity and is primarily presynaptic, dedicated to passing on information, while the somato‐dendritic compartment of the cell is composed of branched smaller diameter neurites that are largely postsynaptic. The establishment of neuronal polarity has until recently mostly been studied *in vitro*, using low‐density cultures of cortical and hippocampal neurons. Under such conditions neuronal polarity first manifests with the emergence of a primary neurite, which extends more rapidly than others and develops into the axon, while the other minor neurites adopt postsynaptic dendritic characteristics. Several signalling pathways, including TGF‐ß, growth factors (e.g. BDNF), LKB and PI3 kinases, have been implicated in bringing about and maintaining asymmetries of the cytoskeleton. Characteristically, axons contain microtubules whose plus ends face away from the cell body, while dendrites have microtubules of mixed (mammals) or opposite polarity (e.g. *Drosophila* and *Caenorhabditis elegans*; for reviews see 18, 19). Perhaps inspired by work from other systems that have associated NADPH oxidase‐generated ROS with the regulation of cell polarisation and growth, for example, *Arabidopsis* hair cell outgrowth 20 and the enforcement of apical dominance in *Aspergillus* hyphae 21, ROS have been investigated as signals regulating the polarisation of neurons. Indeed, *in vitro* studies suggest that ROS produced by NADPH oxidases were required alongside growth factors for the differentiation of neuronal characteristics by PC12 and SH‐SY5Y cells, such as axonal outgrowth 22, 23, 24, 25. *In vivo*, gene expression profiles show that subunits of the NOX2 NADPH oxidase complex are present at the right place and time in mouse and rat embryonic hippocampal neurons 26, 27. In the context of neuronal differentiation mediated by nerve growth factor (NGF), Neuregulin or Retinoic Acid ROS appear to be permissive, acting in parallel to these growth factors. As documented for several growth factors, ROS can enhance downstream kinase signalling by inhibition of phosphatases, whose active sites contain cysteines that are susceptible to oxidation 28. The activity of protein kinases is critical during cellular polarisation, meditating positive feedback loops and signal amplification. Multiple pathways converge onto PI3 kinase, which is enriched at the tip of the future axon as neurons adopt a polarised morphology 29, 30 (reviewed in 18). Interestingly, PI3 kinase signalling can be regulated by ROS, by redox‐mediated inhibition of the phosphatase and tensin homolog (PTEN), which antagonises the PI3 kinase product phosphoinositide‐3‐phosphate 31, 32.

Studies on embryonic hippocampal neurons and cerebellar granule cells in culture have underpinned the idea that ROS contribute to the establishment of neuronal polarity. As cerebellar granule cells differentiate during the first 3 days *in vitro* overall ROS levels increase and become specifically enriched at growth cones and other cytoskeletally dynamic sites. This is paralleled by a ROS‐dependent rise in Tau and MAP2 expression levels, which are representative of axonal and dendritic cytoskeletal specialisation 33. Underlining a requirement for ROS in these processes, neurons derived from NOX2 knockout mice showed reduced neurite length compared to control cells 33. Similarly, in embryonic hippocampal neurons pharmacological or genetic reduction in NADPH oxidase activity, e.g. by expression of the dominant negative DNp22^phox^ regulatory subunit, led to delayed and reduced axon outgrowth 26. Conversely, overactivation of NADPH oxidase by overexpression of the accessory protein p47^phox^ promoted axonal growth 34. These studies suggest that NOX2 activity could indeed contribute to neuronal polarisation and promote neurite outgrowth.

**Figure 1:**
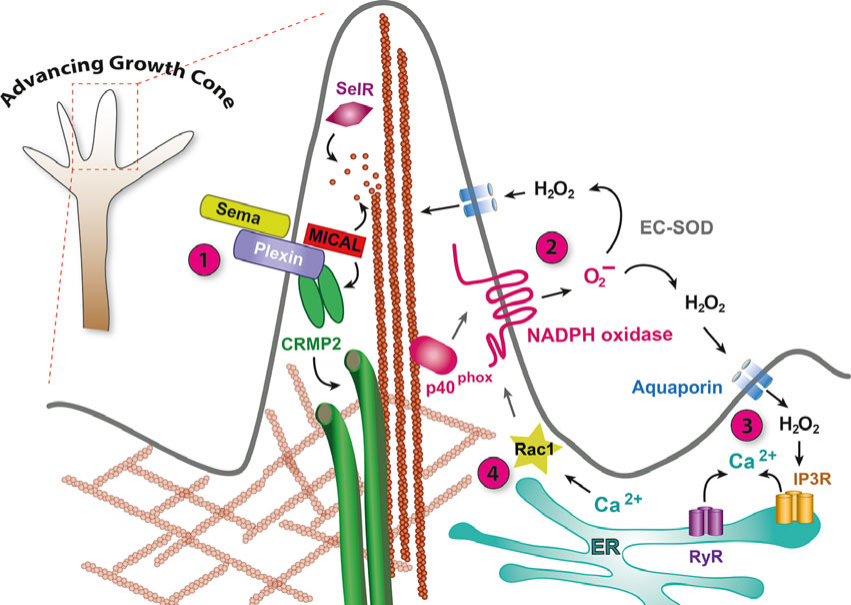
Regulation of the growth cone cytoskeleton by ROS. (1) Semaphorin binding to Plexin activates MICAL monooxygenase. MICAL interactions with F‐actin lead to oxidation of the conserved amino acid Met44 64. Oxidation of actin destabilises F‐actin filaments and promotes binding of the actin severing protein Profilin, thus promoting F‐actin disassembly 67. Oxidised actin monomers have a reduced propensity for polymerisation. MICAL redox activity is opposed by the MsrB enzyme SelR, which specifically reduces Met‐44‐R‐sulfoxide 66. MICAL activation *via* Semaphorin‐Plexin binding also generates H_2_O_2_ that can oxidise CRMP2, enabling it to form a disulfide‐linked homodimer and to transiently interact with Thioredoxin, which stimulates phosphorylation of CRMP2 by glycogen synthase kinase‐3, promoting CRMP2 modification of microtubules and growth cone collapse 71. (2) Superoxide produced by the NOX2 NADPH oxidase catalytic subunit (gp91^phox^) is regulated by translocation of the p40^phox^ regulatory subunit from its association with F‐actin to the plasma membrane upon growth cone engagement with a substrate or guidance cue 58. Converted into hydrogen peroxide by extracellular superoxide dismutase (EC‐SOD), H_2_O_2_ enters the cytoplasm *via* aquaporins and can oxidise cytoskeletal proteins (e.g. F‐actin) 132, 133. (3) H_2_O_2_ also modifies RyRs and IP3Rs triggering release of calcium from internal stores in the ER. (4) Changes in intracellular calcium modify activities of cytoskeletal regulatory proteins, directly or indirectly, e.g. *via* the regulation of Calcium/calmodulin‐dependent kinase II (CamKII) or the phosphatase calcineurin or activation of the protease calpain. Elevated calcium levels also lead to expression of the cytoskeletal and NADPH oxidase regulator Rac1, thus generating a positive feedback loop that can amplify and sustain transient respiratory bursts 34.

The mechanisms by which physiological levels of ROS support neurite outgrowth and axon specification appear to involve release of calcium from intracellular stores, a potent second messenger regulating cytoskeletal organisation and dynamics (reviewed in 35, 36). Short respiratory bursts of NADPH oxidase‐generated ROS promote calcium release by redox modification of ryanodine (RyRs) and inositol‐3‐phosphate receptors (IP3Rs). These in turn lead to increased expression of Rac1, an activator of the NOX2 complex, thus generating a positive feedback loop that can convert initially transient ROS bursts into sustained ROS activation and high levels of intracellular calcium 34; (Fig. 1). However, the extent to which ROS signalling promotes the establishment of neuronal polarity *in vivo* remains to be determined. Phenotypes of NADPH oxidase knockout and RNAi knockdown experiments in several experimental animal models suggest that removal of any one NADPH oxidase still allows the nervous systems to form and function remarkably well, at least to a level sufficient for survival under laboratory conditions 37, 38. This could indicate a degree of functional redundancy. It also suggests that ROS might have a modulatory role rather than being critically required for the establishment of neuronal polarity. This would not be entirely surprising, as during nervous system development neural progenitor cells and their progeny are invariably located within asymmetric environments where local enrichment of numerous cues, including cell adhesion and extracellular matrix proteins, can contribute to cellular polarisation 39, 40, 41 (for review see 18).

### Cytoskeletal modifications by ROS and growth cone pathfinding

Reactive oxygen species can regulate cytoskeletal change at multiple levels; directly *via* redox modification of structural cytoskeletal proteins and indirectly by modification of proteins or signalling pathways that regulate cytoskeletal dynamics. For example, actin and tubulin monomers contain multiple cysteine and methionine residues exposed to the cytoplasm that are subject to redox modifications, notably glutathionylation, nitrosylation and carbonylation 42, 43, 44, 45, 46, 47, 48, 49. Indeed, all major cytoskeletal elements and many cytoskeleton‐associated proteins are subject to direct redox modifications of some kind 50, 51, 52, with substantial fractions of actin, tubulin and neurofilaments found glutathionylated under normal physiological conditions 53. The question of which residues are modified, under what conditions and how this impacts on protein function and dynamics remains live. For alpha‐actin purified from rabbit muscles, *in vitro* and cell culture studies have shown Cys 374 sensitive to glutathionylation, leading to a reduced rate of actin polymerisation and altered actin dynamics 44, 54. Actin Cys 374 glutathionylation has been proposed to occur in response to growth factor and integrin‐stimulated signalling following interactions with the extracellular matrix 54. This actin modification is thought to regulate the disassembly of the actinomyosin complex during cell spreading 52. Along the same lines, disruption of protein deglutathionylation by mutation of glutaredoxin 1 (Grx1), the gene coding for an enzyme that catalyses actin deglutathionylation, led to reduced actin polymerisation and impaired polarisation, chemotaxis, adhesion, and phagocytosis by neutrophils. Conversely, blocking NOX activity led to increased formation of filamentous actin 55. Thus, ROS have increasingly been recognised as important regulators of actin dynamics.

One of the principal ROS sources in neurons is NAPDH oxidases. Their activity is highly regulated, making them prime candidates as regulators of growth cone cytoskeletal dynamics (for reviews see 56 and 57). How NADPH oxidase‐generated ROS regulate cytoskeletal dynamics in neuronal growth cones remains poorly understood. Pharmacological inhibition of NADPH oxidase activity or lowering cytosolic ROS levels led to reduced F‐actin content, retrograde flow and neurite outgrowth. Few studies have documented the localisation of NOX complex components in neuronal growth cones *in vitro*, none as yet *in vivo* 58, 59. In cultured *Aplysia* bag cell neurons the main enzymatic subunit of a NOX2‐type NADPH oxidase, NOX2/gp91^phox^, was seen localised to the plasma membrane, largely distinct from the regulatory subunit p40^phox^ found associated with filopodial actin bundles (Fig. 1). Interestingly, a local stimulus of growth cone interaction with apCAM‐coated beads triggered colocalisation of both subunits to the site of growth cone–substrate interaction. Thus, in growth cones NADPH oxidase subunits are localised to the periphery at sites of actin assembly 58. Their activity is regulated by multiple pathways, including activation following translocation of regulatory subunits, such as p40^phox^ or Rac1, but also by other signalling pathways, such as protein kinase C (PKC) 59. The extent to which local NAPDH oxidase activation regulates growth cone dynamics directly, *via* oxidation of actin and tubulin, or indirectly, through modulation of other signalling pathways, remains to be seen. Both are likely. A study using cultured marsupial kidney cells demonstrated that hydrogen peroxide‐induced chemotaxis and filopodial dynamics were in large part indirectly regulated by ROS *via* local extracellular signal–regulated kinase (ERK) pathway activation, which promoted actin retrograde flow by differential activation and recruitment of cofilin and the Arp2/3 nucleator at the leading edge 60. It is conceivable that ROS signalling in the nervous system might similarly be utilised for noncell autonomous communication, either during the development of synaptic connections or their subsequent adjustment. For example, in a mouse model for multiple sclerosis, persistent activation of NOX2/gp91^phox^ in microglia leads to the impairment of synaptic plasticity in adjacent hippocampal neurons 61.

Thus far, the clearest evidence for post‐translational redox modification of cytoskeletal proteins directing growth cone pathfinding derives from studies of Semaphorin‐Plexin signalling. The cytoplasmic tail of Plexin interacts with the NADPH‐dependent monooxygenase, molecule interacting with CasL (MICAL), which is activated upon binding of Semaphorin guidance cues 62, 63, 64, 65, 66, 67 (for review see 68; Fig. 1). Elegant experiments by the Terman laboratory and collaborators demonstrated that the amino‐terminal NADPH‐dependent redox domain of MICAL‐1 binds F‐actin and directly oxidises the methionine residues Met44 and Met47 64, 65. These redox modifications are regulated and can be reversed by a methionine sulfoxide reductase, (MsrB/SelR), which catalyses the reduction of methionine sulfoxide to methionine 66. MICAL‐1‐mediated oxidation of actin weakens inter‐actin contacts while simultaneously increasing the binding affinity of the F‐actin severing protein cofilin by more than an order of magnitude. This synergistic effect of actin destabilisation by oxidation and concomitantly increased binding of cofilin promotes F‐actin disassembly. In addition, MICAL‐mediated oxidation decreases the capacity of actin for repolymerisation, thus further impacting on the dynamics of the actin cytoskeleton 67. Thus, Semaphorin‐Plexin guidance cue–receptor interactions are directly transduced to redox modification of the actin cytoskeleton, leading to F‐actin disassembly and altered reassembly dynamics.

In addition, MICAL activation also impacts on the microtubule cytoskeleton *via* another binding partner, collapsin response mediator protein 2 (CRMP2). CRMP2 is responsible for Semaphorin‐induced growth cone collapse 69 and interacts with tubulin heterodimers regulating microtubule dynamics 1 62, 70. Current data suggest that MICAL‐1 binds CRMP2, and that upon MICAL monooxygenase activation (e.g. by Semaphorin binding to Plexin), hydrogen peroxide is produced that oxidises CRMP2. Oxidation of CRMP2 promotes formation of disulfide‐linked CRMP2 homodimers, which interact with thioredoxin, which in turn promotes their phosphorylation by GSK‐3ß, microtubule disassembly and growth cone collapse 71; (Fig. 1). In summary, direct redox modifications of cytoskeletal elements, notably actin and tubulin, as well as of regulators of cytoskeletal dynamics, such as CRMP2, lie at the heart of Semaphorin‐Plexin growth cone guidance. It is conceivable that redox modification of cytoskeletal proteins extends to other guidance cue signalling pathways, either directly or in a modulatory capacity where the cellular redox state determines growth cone responses to extracellular cues.

## Connectivity and structural plasticity

For decades ROS have been implicated in neurodegenerative conditions, largely thought of as destructive agents 72, 73. Increasingly ROS have also been viewed as regulators and modulators of signalling pathways and gene expression, of which many are known to regulate neuronal growth and plasticity (reviewed in 56, 57, 74). Several years ago we provided the first direct *in vivo* evidence of ROS as regulators of synaptic terminal growth, under pathological conditions in an experimental animal model (*Drosophila*) for lysosomal storage disease 75. The study demonstrated that oxidative stress resulting from lysosomal storage dysfunction led to activation of the JNK cascade and activation of the immediate early genes c‐Jun and c‐Fos (AP‐1) which in turn led to altered growth of neuromuscular junction terminals 75. Along with a prior study by Sanyal and colleagues 76, this work identified AP‐1 as the major adaptive response to ROS in neurons. The JNK/AP‐1 signalling pathway has long been known to be critical in many neuronal functions and is a well‐known mediator of synaptic and oxidative stress responses (reviewed in 74). AP‐1 is a heterodimer composed of the leucine‐zipper transcription factors Fos and Jun. Fos is one of the major immediate early transcription factors mediating long‐term synaptic changes during long‐term potentiation (LTP) 77, 78, although the actual mechanism inducing JNK phosphorylation and subsequent AP‐1 activation in this process remains obscure. Activation of JNK/AP‐1 by oxidative stress is thought to reinforce autophagy, and many genes encoding autophagy proteins are direct transcriptional targets of AP‐1 79. Some evidence suggests that activation of autophagy *via* the oxidative stress‐induced JNK/AP‐1 pathway can regulate synaptic terminal size and strength at the *Drosophila* larval neuromuscular synapse 75, 76, 80. The data point to the importance of the JNK/AP‐1 pathway as regulating synaptic function and to ROS as critical upstream signals during normal physiological conditions as well as under oxidative stress.

In a follow‐up study we since asked whether ROS also act as regulators of synaptic terminal growth and plasticity under normal physiological conditions, which until now has remained largely unexplored. Indeed, we found that ROS, in particular hydrogen peroxide, are necessary for activity‐induced synaptic terminal growth and are sufficient to drive synaptic terminal growth 81. Specifically, overactivation of motoneurons leads to increased mitochondrial ROS levels at the presynaptic neuromuscular junction of *Drosophila* larvae, previously also reported for cultured hippocampal neurons 82, 83. In *Drosophila* larvae, activity‐generated ROS promote altered synaptic terminal growth, generating more, albeit smaller synaptic varicosities (boutons) along with a reduction in the number of synaptic release sites. Postsynaptic dendrites similarly undergo homeostatic structural adjustments in response to activity‐generated ROS, leading to smaller dendritic arbours, which we previously showed equates to reduced synaptic input sites and reduced synaptic drive 84. The signalling and downstream effector pathways of neuronal activity‐generated ROS are only just being sketched out. We found that neuronal ROS signal *via* the conserved redox‐sensitive protein DJ‐1ß, a homologue of vertebrate DJ‐1 (PARK7) 85, 86, which appears to act as a neuronal redox sensor. Oxidation of DJ‐1ß increases inhibitory interactions with the phosphatase PTEN thus leading to disinhibition of PI3kinase signalling, a known regulator of synaptic terminal growth 87, 88, 89, 90. Whether changes in dendritic growth are also regulated by PTEN‐PI3kinase as a ROS effector pathway, and how synaptic terminal growth might be coupled to synaptic connectivity remain to be determined. Importantly, ROS are obligate signals for activity‐regulated structural plasticity of synaptic terminals, either instructive or permissive. Targeted abrogation of neuronal ROS signalling in *Drosophila* larval motoneurons, by expression of a modified form of the DJ‐1β redox sensor (the conserved Cysteine 106 mutated to a nonoxidisable Alanine 86, 91) prevented the locomotor network from adjusting homeostatically in response to increased levels of network activity, resulting in abnormal motor output 81.

Precisely how neuronal activation leads to the generation of ROS signals is not clear and is likely context specific. In general, ROS are formed as obligate by‐products of mitochondrial respiratory ATP synthesis, by ‘leakage’ of the electron transport chain 72. Thus, mitochondrial ROS could potentially provide neurons with a readout of their energetic demand. In addition, NMDA receptor stimulation has been shown to trigger ROS generation by either mitochondria 92, 93 or NADPH oxidases 94. NADPH oxidase activity is subject to complex regulatory pathways, of which many are associated with neuronal activation, such as elevated intracellular calcium levels, Protein kinases C and A, as well as calmodulin and calcium/calmodulin‐dependent kinase II (CamKII) 34, 56, 95, 96, 97, 98.

It has become increasingly evident that during normal nervous system development and function ROS act as second messengers that regulate multiple aspects, from neuronal polarity and axon growth cone behaviour to structural plasticity. As such ROS have more recently been investigated as potentially involved in the aetiology of psychiatric disorders with neurodevelopmental origins. For example, in mammalian brains cortical parvalbumin‐expressing inhibitory interneurons (PVIs) are critical to the excitation–inhibition balance and therefore function of many cortical networks. These fast‐spiking neurons are arguably highly susceptible to ROS generated by mitochondrial ATP metabolism and it is thought that their surrounding perineuronal nets confer protection of the PVIs to oxidative challenges 99. Imbalances in ROS metabolism and buffering are thought of as potentially impacting on the development of cortical networks during so‐called critical periods, leading to sub‐optimal network performance and neuropsychiatric disorders 100. Supporting this hypothesis are reports of *post mortem* brain tissue from patients with schizophrenia, bipolar or autism spectrum disorder exhibiting reductions in cortical PVIs 101, 102, 103. Moreover, a recent study did indeed find that conditions of elevated oxidative stress preceded and correlated with reduced integrity of PVIs in mouse models for these disorders 104.

## Synaptic transmission and plasticity

Synaptic plasticity describes the ability of synapses to adjust their strength, connectivity and structure in response to previously experienced activity. The inherent plasticity of neurons is key to neuronal network development and in networks allows for adaptation, memory and learning. Synaptic strength may be enhanced or reduced depending upon the neuronal context and the nature of stimulation, the best‐studied examples being LTP and long‐term depression (LTD). LTP was originally described following repetitive stimulation of the perforant path fibres to the dentate area of the hippocampus 105. The high‐frequency stimulation (HFS) used for induction of LTP results in opening of NMDA receptors and thus elevated intracellular Ca^2+^. This leads to the adjustment of synaptic strength *via* direct and transcriptionally regulated modification of synaptic proteins, and changes in the composition of synaptic protein complexes (for review see 57, 106).

**Figure 2:**
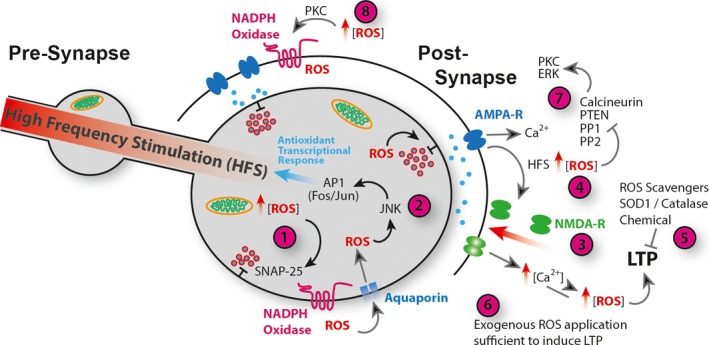
ROS regulation of Synaptic Plasticity. (1) Presynaptic ROS, derived from mitochondria or NADPH oxidase activity regulates vesicle release *via* oxidation of SNAP‐25 124. ROS regulate release probabilities with subsynaptic terminal resolution 122, 123. (2) Increases in ROS lead to activation of JNK and AP‐1, which promote expression of antioxidant encoding genes 14 and others required for autophagy 79. AP‐1 mediates neuronal adaptive responses to ROS 75, 76. (3) Postsynaptic LTP, in response to HFS, drives recruitment and opening of NMDA receptors and consequent elevation of intracellular Ca^2+^ concentration. HFS causes elevated ROS production and a shift towards an oxidative environment in the synaptic terminal (4) 83, 92. HFS‐induced LTP requires ROS (5) 108 and exogenous application of ROS (6) is sufficient to induce LTP in the absence of HFS 111. ROS regulate canonical synaptic plasticity pathways *via* direct oxidative modification, and inhibition of phosphatases PP1, PP2, PTEN and Calcineurin resulting in increased kinase signalling including ERK and PKC (7) 114, 115, 116, 117, 118, 119, 120, 121. Also, ROS‐activated PKC stimulates NADPH‐oxidase activation and exacerbated ROS production (8) 107, 111.

A role for ROS in synaptic plasticity has been demonstrated in various model systems and areas of the nervous system (Fig. 2). ROS production is elevated in hippocampal slice preparations following increased neuronal activity, NMDA receptor activation and subsequent LTP 92. In mouse hippocampus NMDA receptor activation triggers ROS generation through the NOX2 NADPH oxidase, regulated by PKC 94. Importantly, acute application of cell permeable superoxide scavengers can block HFS‐induced LTP in hippocampal slices. Dysregulation of ROS *via* transgenic mis‐expression of SOD1 or Catalase similarly blocked LTP, suggesting that LTP requires ROS and at the same time is sensitive to the cellular redox state 107, 108, 109, 110. Conversely, bath applied elevation of ROS in hippocampal slices can be sufficient to induce LTP in the CA1 region 111. ROS are also required and sufficient for the induction and maintenance of spinal cord LTP, contributing to central sensitisation and chronic neuropathic pain 112. Interestingly, in cerebellar Purkinje neurons superoxide is required for LTD, although in these cells synaptic depression (as opposed to potentiation) requires elevated intracellular calcium concentration 113.

The specific protein targets of ROS for regulating synaptic plasticity and the interplay between Ca^2+^ and ROS‐regulated pathways are exciting and thriving research fields 57. In particular, the link between ROS and Ca^2+^‐regulated plasticity signalling pathways raises the possibility that synaptic redox state provides a permissive environment that, depending upon context, positively or negatively tunes neuronal plasticity in response to Ca^2+^ signalling. One of the recurrent challenges is to identify the mechanisms through which ROS act, whether these are direct redox modification of effector proteins, or whether ROS impact on cellular function indirectly by modulating other signalling pathways. For example, several protein phosphatases (PP), including PP1 and PP2, PTEN and calcineurin (PP 2B), are regulated by ROS, perhaps suggesting a general role for ROS as negative regulators of kinase cascade pathways 114, 115, 116, 117, 118, 119. In the capacity of phosphatase inhibitors ROS have modulatory access to pathways known to regulate synaptic plasticity. Indeed, in response to H_2_O_2_ application the phosphorylation state of ERK is upregulated in hippocampal slices, cortical neurons and PC12 cells 120, 121. Similarly, activation of PKC, which is required for hippocampal LTP, is triggered by ROS 111 (Fig. 2).

Direct regulation of synaptic function by redox modification of synaptic proteins also appears likely. For example, exposure of frog NMJs to H_2_O_2_ revealed their capability to directly regulate synaptic release probabilities. While synaptic strength remained unchanged, the authors observed altered quantal release synchronicity when comparing proximal vs. distal parts of the NMJ, suggesting a modulatory role for ROS or redox state with subsynaptic resolution 122, 123. In a follow‐up study the same group identified SNAP‐25, a component of the SNARE complex and regulator of synaptic vesicle fusion, as a direct ROS target, whose oxidation by H_2_O_2_ mediated downregulation of release synchronicity 124. Excitingly, a recent study demonstrated a role for targeted redox modifications in regulating presynaptic homeostatic adjustments of quantal content. Postsynaptic Semaphorin to presynaptic Plexin signalling activates the MICAL monooxygenase. This is known to specifically modify the actin cytoskeleton, which might impact on the presynaptic cytoskeleton in general and actin‐mediated vesicle tethering in particular 125 (Fig. 2).

In summary, current evidence suggests that synaptic plasticity is regulated by both direct and indirect modes of ROS action. It will almost certainly be determined by context, such as local redox state of pre‐ or postsynaptic sites, the nature (e.g. hydrogen peroxide vs. superoxide vs. nitric oxide), source and subcellular localisation of ROS (e.g. mitochondrial vs. plasma membrane localised NADPH oxidase vs. cytoskeleton associated monooygenase). In addition, different protein modifications might be subject to distinct ROS concentration thresholds, which could endow ROS signalling with a further degree of flexibility and complexity.

## Conclusions

A large body of work has demonstrated the importance of ROS as signals that regulate numerous processes during neuronal development and nervous system function. With the majority of work to date having been carried out in cultured cells, it will be important to verify these by studying ROS signalling *in vivo*. While whole animal knockout mutants, for example, of NADPH oxidases, are informative, future work would ideally use cell‐specific mosaic manipulations with which to unambiguously determine cell type specificity of ROS requirements. One central aspect will be to determine how ROS generation is regulated, for example, the subcellular localisation of NADPH oxidases and other ROS generators. Previous studies have reported cue‐dependent changes in regulatory subunits as mechanisms for controlling ROS production 58, 126. Equally important for our understanding will be genetically encoded tools for visualising ROS and cellular redox potential, ideally with specificity for different ROS species 127, 128, 129, 130. Under normal physiological conditions ROS signals are expected to be confined in space and time. In the nervous system, in particular, there is a need for new reagents that will allow genetically targeted *in vivo* manipulation of ROS generators and scavengers with spatial and temporal control appropriate for studying neuronal cell behaviour and synaptic transmission. Following the success of optogenetic modulators of neuronal excitability, this might take the route for engineering optogenetic solutions for ROS generation and sequestration 131.

## References

[1] Wang W , Fang H , Groom L , Cheng A , Zhang W , Liu J , Wang X , Li K , Han P , Zheng M *et al* (2008) Superoxide flashes in single mitochondria. Cell 134, 279–290. 18662543 10.1016/j.cell.2008.06.017 PMC2547996

[2] Gross E , Sevier CS , Heldman N , Vitu E , Bentzur M , Kaiser CA , Thorpe C and Fass D (2006) Generating disulfides enzymatically: reaction products and electron acceptors of the endoplasmic reticulum thiol oxidase Ero1p. Proc Natl Acad Sci USA 103, 299–304. 16407158 10.1073/pnas.0506448103 PMC1326156

[3] Boveris A and Chance B (1973) The mitochondrial generation of hydrogen peroxide. General properties and effect of hyperbaric oxygen. Biochem J 134, 707–716. 4749271 10.1042/bj1340707 PMC1177867

[4] Kukreja RC , Kontos HA , Hess ML and Ellis EF (1986) PGH synthase and lipoxygenase generate superoxide in the presence of NADH or NADPH. Circ Res 59, 612–619. 3028671 10.1161/01.res.59.6.612

[5] O'Donnell VB and Azzi A (1996) High rates of extracellular superoxide generation by cultured human fibroblasts: involvement of a lipid‐metabolizing enzyme. Biochem J 318 (Pt 3), 805–812. 8836123 10.1042/bj3180805 PMC1217690

[6] McNally JS , Davis ME , Giddens DP , Saha A , Hwang J , Dikalov S , Jo H and Harrison DG (2003) Role of xanthine oxidoreductase and NAD(P)H oxidase in endothelial superoxide production in response to oscillatory shear stress. Am J Physiol Heart Circ Physiol 285, H2290–H2297. 12958034 10.1152/ajpheart.00515.2003

[7] Spina MB and Cohen G (1989) Dopamine turnover and glutathione oxidation: implications for Parkinson disease. Proc Natl Acad Sci USA 86, 1398–1400. 2919185 10.1073/pnas.86.4.1398 PMC286698

[8] Martins RN , Harper CG , Stokes GB and Masters CL (1986) Increased cerebral glucose‐6‐phosphate dehydrogenase activity in Alzheimer's disease may reflect oxidative stress. J Neurochem 46, 1042–1045. 3950618 10.1111/j.1471-4159.1986.tb00615.x

[9] Höhn A and Grune T (2013) Lipofuscin: formation, effects and role of macroautophagy. Redox Biol 1, 140–144. 24024146 10.1016/j.redox.2013.01.006 PMC3757681

[10] Ahlgren‐Beckendorf JA , Reising AM , Schander MA , Herdler JW and Johnson JA (1999) Coordinate regulation of NAD(P)H:quinone oxidoreductase and glutathione‐S‐transferases in primary cultures of rat neurons and glia: role of the antioxidant/electrophile responsive element. Glia 25, 131–142. 9890628

[11] Shih AY , Johnson DA , Wong G , Kraft AD , Jiang L , Erb H , Johnson JA and Murphy TH (2003) Coordinate regulation of glutathione biosynthesis and release by Nrf2‐expressing glia potently protects neurons from oxidative stress. J Neurosci 23, 3394–3406. 12716947 10.1523/JNEUROSCI.23-08-03394.2003 PMC6742304

[12] Jimenez‐Blasco D , Santofimia‐Castaño P , González A , Almeida A and Bolaños JP (2015) Astrocyte NMDA receptors' activity sustains neuronal survival through a Cdk5‐Nrf2 pathway. Cell Death Differ 22, 1877–1889. 25909891 10.1038/cdd.2015.49 PMC4648333

[13] Longo N (2009) Disorders of biopterin metabolism. J Inherit Metab Dis 32, 333–342. 19234759 10.1007/s10545-009-1067-2

[14] Baxter PS and Hardingham GE (2016) Adaptive regulation of the brain's antioxidant defences by neurons and astrocytes. Free Radic Biol Med 100, 147–152. 27365123 10.1016/j.freeradbiomed.2016.06.027 PMC5145800

[15] Baxter PS , Bell KFS , Hasel P , Kaindl AM , Fricker M , Thomson D , Cregan SP , Gillingwater TH and Hardingham GE (2015) Synaptic NMDA receptor activity is coupled to the transcriptional control of the glutathione system. Nat Commun 6, 6761. 25854456 10.1038/ncomms7761 PMC4403319

[16] Papadia S , Soriano FX , Léveillé F , Martel M‐A , Dakin KA , Hansen HH , Kaindl A , Sifringer M , Fowler J , Stefovska V *et al* (2008) Synaptic NMDA receptor activity boosts intrinsic antioxidant defenses. Nat Neurosci 11, 476–487. 18344994 10.1038/nn2071 PMC2556874

[17] Bell KFS , Al‐Mubarak B , Martel M‐A , Mckay S , Wheelan N , Hasel P , Márkus NM , Baxter P , Deighton RF , Serio A *et al* (2015) Neuronal development is promoted by weakened intrinsic antioxidant defences due to epigenetic repression of Nrf2. Nat Commun 6, 7066. 25967870 10.1038/ncomms8066 PMC4441249

[18] Yogev S and Shen K (2017) Establishing neuronal polarity with environmental and intrinsic mechanisms. Neuron 96, 638–650. 29096077 10.1016/j.neuron.2017.10.021

[19] Schelski M and Bradke F (2017) Neuronal polarization: from spatiotemporal signaling to cytoskeletal dynamics. Mol Cell Neurosci 84, 11–28. 28363876 10.1016/j.mcn.2017.03.008

[20] Foreman J , Demidchik V , Bothwell JHF , Mylona P , Miedema H , Torres MA , Linstead P , Costa S , Brownlee C , Jones JDG *et al* (2003) Reactive oxygen species produced by NADPH oxidase regulate plant cell growth. Nature 422, 442–446. 12660786 10.1038/nature01485

[21] Semighini CP and Harris SD (2008) Regulation of apical dominance in Aspergillus nidulans hyphae by reactive oxygen species. Genetics 179, 1919–1932. 18689883 10.1534/genetics.108.089318 PMC2516069

[22] Suzukawa K , Miura K , Mitsushita J , Resau J , Hirose K , Crystal R and Kamata T (2000) Nerve growth factor‐induced neuronal differentiation requires generation of Rac1‐regulated reactive oxygen species. J Biol Chem 275, 13175–13178. 10788420 10.1074/jbc.275.18.13175

[23] Goldsmit Y , Erlich S and Pinkas‐Kramarski R (2001) Neuregulin induces sustained reactive oxygen species generation to mediate neuronal differentiation. Cell Mol Neurobiol 21, 753–769. 12043846 10.1023/A:1015108306171 PMC11533816

[24] Kamata H , Oka S‐I , Shibukawa Y , Kakuta J and Hirata H (2005) Redox regulation of nerve growth factor‐induced neuronal differentiation of PC12 cells through modulation of the nerve growth factor receptor, TrkA. Arch Biochem Biophys 434, 16–25. 15629104 10.1016/j.abb.2004.07.036

[25] Nitti M , Furfaro AL , Cevasco C , Traverso N , Marinari UM , Pronzato MA and Domenicotti C (2010) PKC delta and NADPH oxidase in retinoic acid‐induced neuroblastoma cell differentiation. Cell Signal 22, 828–835. 20074641 10.1016/j.cellsig.2010.01.007

[26] Wilson C , Núñez MT and González‐Billault C (2015) Contribution of NADPH oxidase to the establishment of hippocampal neuronal polarity in culture. J Cell Sci 128, 2989–2995. 26101350 10.1242/jcs.168567

[27] Tejada‐Simon MV , Serrano F , Villasana LE , Kanterewicz BI , Wu GY , Quinn MT and Klann E (2005) Synaptic localization of a functional NADPH oxidase in the mouse hippocampus. Mol Cell Neurosci 29, 97–106. 15866050 10.1016/j.mcn.2005.01.007 PMC2013304

[28] Tonks NK (2005) Redox redux: revisiting PTPs and the control of cell signaling. Cell 121, 667–670. 15935753 10.1016/j.cell.2005.05.016

[29] Ménager C , Arimura N , Fukata Y and Kaibuchi K (2004) PIP3 is involved in neuronal polarization and axon formation. J Neurochem 89, 109–118. 15030394 10.1046/j.1471-4159.2004.02302.x

[30] Shi S‐H , Jan LY and Jan YN (2003) Hippocampal neuronal polarity specified by spatially localized mPar3/mPar6 and PI 3‐kinase activity. Cell 112, 63–75. 12526794 10.1016/s0092-8674(02)01249-7

[31] Kim RH , Peters M , Jang Y , Shi W , Pintilie M , Fletcher GC , DeLuca C , Liepa J , Zhou L , Snow B *et al* (2005) DJ‐1, a novel regulator of the tumor suppressor PTEN. Cancer Cell 7, 263–273. 15766664 10.1016/j.ccr.2005.02.010

[32] Kim Y‐C , Kitaura H , Taira T , Iguchi‐Ariga SMM and Ariga H (2009) Oxidation of DJ‐1‐dependent cell transformation through direct binding of DJ‐1 to PTEN. Int J Oncol 35, 1331–1341. 19885556

[33] Olguín‐Albuerne M and Morán J (2015) ROS produced by NOX2 control *in vitro* development of cerebellar granule neurons development. ASN Neuro 7, 1–28. 10.1177/1759091415578712 PMC4720178 25873309

[34] Wilson C , Muñoz‐Palma E , Henríquez DR , Palmisano I , Núñez MT , Di Giovanni S and González‐Billault C (2016) A feed‐forward mechanism involving the NOX complex and RyR‐mediated Ca2+ release during axonal specification. J Neurosci 36, 11107–11119. 27798190 10.1523/JNEUROSCI.1455-16.2016 PMC6705650

[35] Akiyama H and Kamiguchi H (2013) Second messenger networks for accurate growth cone guidance. Dev Neurobiol 75, 411–422. 24285606 10.1002/dneu.22157

[36] Gasperini RJ , Pavez M , Thompson AC , Mitchell CB , Hardy H , Young KM , Chilton JK and Foa L (2017) How does calcium interact with the cytoskeleton to regulate growth cone motility during axon pathfinding? Mol Cell Neurosci 84, 29–35. 28765051 10.1016/j.mcn.2017.07.006

[37] Aguirre J and Lambeth JD (2010) Nox enzymes from fungus to fly to fish and what they tell us about Nox function in mammals. Free Radic Biol Med 49, 1342–1353. 20696238 10.1016/j.freeradbiomed.2010.07.027 PMC2981133

[38] Sirokmány G , Donkó Á and Geiszt M (2016) Nox/Duox family of NADPH oxidases: lessons from knockout mouse models. Trends Pharmacol Sci 37, 318–327. 26861575 10.1016/j.tips.2016.01.006

[39] Randlett O , Poggi L , Zolessi FR and Harris WA (2011) The oriented emergence of axons from retinal ganglion cells is directed by laminin contact in vivo. Neuron 70, 266–280. 21521613 10.1016/j.neuron.2011.03.013 PMC3087191

[40] Desai RA , Gao L , Raghavan S , Liu WF and Chen CS (2009) Cell polarity triggered by cell‐cell adhesion via E‐cadherin. J Cell Sci 122, 905–911. 19258396 10.1242/jcs.028183 PMC2720926

[41] McNeill H , Ozawa M , Kemler R and Nelson WJ (1990) Novel function of the cell adhesion molecule uvomorulin as an inducer of cell surface polarity. Cell 62, 309–316. 2164888 10.1016/0092-8674(90)90368-o

[42] Dalle‐Donne I , Rossi R , Giustarini D , Gagliano N , Lusini L , Milzani A , Di Simplicio P and Colombo R (2001) Actin carbonylation: from a simple marker of protein oxidation to relevant signs of severe functional impairment. Free Radic Biol Med 31, 1075–1083. 11677040 10.1016/s0891-5849(01)00690-6

[43] Dalle‐Donne I , Rossi R , Giustarini D , Gagliano N , Di Simplicio P , Colombo R and Milzani A (2002) Methionine oxidation as a major cause of the functional impairment of oxidized actin. Free Radic Biol Med 32, 927–937. 11978495 10.1016/s0891-5849(02)00799-2

[44] Dalle‐Donne I , Giustarini D , Rossi R , Colombo R and Milzani A (2003) Reversible S‐glutathionylation of Cys 374 regulates actin filament formation by inducing structural changes in the actin molecule. Free Radic Biol Med 34, 23–32. 12498976 10.1016/s0891-5849(02)01182-6

[45] Dalle‐Donne I , Rossi R , Milzani A , Di Simplicio P and Colombo R (2001) The actin cytoskeleton response to oxidants: from small heat shock protein phosphorylation to changes in the redox state of actin itself. Free Radic Biol Med 31, 1624–1632. 11744337 10.1016/s0891-5849(01)00749-3

[46] Chai YC , Ashraf SS , Rokutan K , Johnston RB and Thomas JA (1994) S‐thiolation of individual human neutrophil proteins including actin by stimulation of the respiratory burst: evidence against a role for glutathione disulfide. Arch Biochem Biophys 310, 273–281. 8161216 10.1006/abbi.1994.1167

[47] Landino LM , Hasan R , McGaw A , Cooley S , Smith AW , Masselam K and Kim G (2002) Peroxynitrite oxidation of tubulin sulfhydryls inhibits microtubule polymerization. Arch Biochem Biophys 398, 213–220. 11831852 10.1006/abbi.2001.2729

[48] Landino LM , Moynihan KL , Todd JV and Kennett KL (2004) Modulation of the redox state of tubulin by the glutathione/glutaredoxin reductase system. Biochem Biophys Res Commun 314, 555–560. 14733943 10.1016/j.bbrc.2003.12.126

[49] Lassing I , Schmitzberger F , Björnstedt M , Holmgren A , Nordlund P , Schutt CE and Lindberg U (2007) Molecular and structural basis for redox regulation of beta‐actin. J Mol Biol 370, 331–348. 17521670 10.1016/j.jmb.2007.04.056

[50] Lind C , Gerdes R , Hamnell Y , Schuppe‐Koistinen I , von Löwenhielm HB , Holmgren A and Cotgreave IA (2002) Identification of S‐glutathionylated cellular proteins during oxidative stress and constitutive metabolism by affinity purification and proteomic analysis. Arch Biochem Biophys 406, 229–240. 12361711 10.1016/s0003-9861(02)00468-x

[51] Fratelli M , Demol H , Puype M , Casagrande S , Eberini I , Salmona M , Bonetto V , Mengozzi M , Duffieux F , Miclet E *et al* (2002) Identification by redox proteomics of glutathionylated proteins in oxidatively stressed human T lymphocytes. Proc Natl Acad Sci USA 99, 3505–3510. 11904414 10.1073/pnas.052592699 PMC122553

[52] Fiaschi T , Cozzi G , Raugei G , Formigli L , Ramponi G and Chiarugi P (2006) Redox regulation of beta‐actin during integrin‐mediated cell adhesion. J Biol Chem 281, 22983–22991. 16757472 10.1074/jbc.M603040200

[53] Sparaco M , Gaeta LM , Tozzi G , Bertini E , Pastore A , Simonati A , Santorelli FM and Piemonte F (2006) Protein glutathionylation in human central nervous system: potential role in redox regulation of neuronal defense against free radicals. J Neurosci Res 83, 256–263. 16385584 10.1002/jnr.20729

[54] Wang J , Boja ES , Tan W , Tekle E , Fales HM , English S , Mieyal JJ and Chock PB (2001) Reversible glutathionylation regulates actin polymerization in A431 cells. J Biol Chem 276, 47763–47766. 11684673 10.1074/jbc.C100415200

[55] Sakai J , Li J , Subramanian KK , Mondal S , Bajrami B , Hattori H , Jia Y , Dickinson BC , Zhong J , Ye K *et al* (2012) Reactive oxygen species‐induced actin glutathionylation controls actin dynamics in neutrophils. Immunity 37, 1037–1049. 23159440 10.1016/j.immuni.2012.08.017 PMC3525814

[56] Massaad CA and Klann E (2011) Reactive oxygen species in the regulation of synaptic plasticity and memory. Antioxid Redox Signal 14, 2013–2054. 20649473 10.1089/ars.2010.3208 PMC3078504

[57] Hidalgo C and Arias‐Cavieres A (2016) Calcium, reactive oxygen species, and synaptic plasticity. Physiology (Bethesda) 31, 201–215. 27053734 10.1152/physiol.00038.2015

[58] Munnamalai V , Weaver CJ , Weisheit CE , Venkatraman P , Agim ZS , Quinn MT and Suter DM (2014) Bidirectional interactions between NOX2‐type NADPH oxidase and the F‐actin cytoskeleton in neuronal growth cones. J Neurochem 130, 526–540. 24702317 10.1111/jnc.12734 PMC4126878

[59] Munnamalai V and Suter DM (2009) Reactive oxygen species regulate F‐actin dynamics in neuronal growth cones and neurite outgrowth. J Neurochem 108, 644–661. 19054285 10.1111/j.1471-4159.2008.05787.x PMC2995541

[60] Taulet N , Delorme‐Walker VD and DerMardirossian C (2012) Reactive oxygen species regulate protrusion efficiency by controlling actin dynamics. PLoS ONE 7, e41342. 22876286 10.1371/journal.pone.0041342 PMC3410878

[61] Di Filippo M , de Iure A , Giampà C , Chiasserini D , Tozzi A , Orvietani PL , Ghiglieri V , Tantucci M , Durante V , Quiroga‐Varela A *et al* (2016) Persistent activation of microglia and NADPH oxidase [corrected] drive hippocampal dysfunction in experimental multiple sclerosis. Sci Rep 6, 20926. 26887636 10.1038/srep20926 PMC4757867

[62] Schmidt EF , Shim S‐O and Strittmatter SM (2008) Release of MICAL autoinhibition by semaphorin‐plexin signaling promotes interaction with collapsin response mediator protein. J Neurosci 28, 2287–2297. 18305261 10.1523/JNEUROSCI.5646-07.2008 PMC2846290

[63] Terman JR , Mao T , Pasterkamp RJ , Yu H‐H and Kolodkin AL (2002) MICALs, a family of conserved flavoprotein oxidoreductases, function in plexin‐mediated axonal repulsion. Cell 109, 887–900. 12110185 10.1016/s0092-8674(02)00794-8

[64] Hung R‐J , Pak CW and Terman JR (2011) Direct redox regulation of F‐actin assembly and disassembly by Mical. Science 334, 1710–1713. 22116028 10.1126/science.1211956 PMC3612955

[65] Hung R‐J , Yazdani U , Yoon J , Wu H , Yang T , Gupta N , Huang Z , van Berkel WJH and Terman JR (2010) Mical links semaphorins to F‐actin disassembly. Nature 463, 823–827. 20148037 10.1038/nature08724 PMC3215588

[66] Hung R‐J , Spaeth CS , Yesilyurt HG and Terman JR (2013) SelR reverses Mical‐mediated oxidation of actin to regulate F‐actin dynamics. Nat Cell Biol 15, 1445–1454. 24212093 10.1038/ncb2871 PMC4254815

[67] Grintsevich EE , Yesilyurt HG , Rich SK , Hung R‐J , Terman JR and Reisler E (2016) F‐actin dismantling through a redox‐driven synergy between Mical and cofilin. Nat Cell Biol 18, 876–885. 27454820 10.1038/ncb3390 PMC4966907

[68] Zhou Y , Gunput R‐AF , Adolfs Y and Pasterkamp RJ (2011) MICALs in control of the cytoskeleton, exocytosis, and cell death. Cell Mol Life Sci 68, 4033–4044. 21822644 10.1007/s00018-011-0787-2 PMC3221843

[69] Goshima Y , Nakamura F , Strittmatter P and Strittmatter SM (1995) Collapsin‐induced growth cone collapse mediated by an intracellular protein related to UNC‐33. Nature 376, 509–514. 7637782 10.1038/376509a0

[70] Fukata Y , Itoh TJ , Kimura T , Ménager C , Nishimura T , Shiromizu T , Watanabe H , Inagaki N , Iwamatsu A , Hotani H *et al* (2002) CRMP‐2 binds to tubulin heterodimers to promote microtubule assembly. Nat Cell Biol 4, 583–591. 12134159 10.1038/ncb825

[71] Morinaka A , Yamada M , Itofusa R , Funato Y , Yoshimura Y , Nakamura F , Yoshimura T , Kaibuchi K , Goshima Y , Hoshino M *et al* (2011) Thioredoxin mediates oxidation‐dependent phosphorylation of CRMP2 and growth cone collapse. Sci Signal 4, ra26. 21521879 10.1126/scisignal.2001127

[72] Halliwell B (1992) Reactive oxygen species and the central nervous system. J Neurochem 59, 1609–1623. 1402908 10.1111/j.1471-4159.1992.tb10990.x

[73] Wang X and Michaelis EK (2010) Selective neuronal vulnerability to oxidative stress in the brain. Front Aging Neurosci 2, 12. 20552050 10.3389/fnagi.2010.00012 PMC2874397

[74] Milton VJ and Sweeney ST (2012) Oxidative stress in synapse development and function. Dev Neurobiol 72, 100–110. 21793225 10.1002/dneu.20957

[75] Milton VJ , Jarrett HE , Gowers K , Chalak S , Briggs L , Robinson IM and Sweeney ST (2011) Oxidative stress induces overgrowth of the *Drosophila* neuromuscular junction. Proc Natl Acad Sci USA 108, 17521–17526. 21987827 10.1073/pnas.1014511108 PMC3198377

[76] Sanyal S , Sandstrom DJ , Hoeffer CA and Ramaswami M (2002) AP‐1 functions upstream of CREB to control synaptic plasticity in *Drosophila* . Nature 416, 870–874. 11976688 10.1038/416870a

[77] Fleischmann A , Hvalby O , Jensen V , Strekalova T , Zacher C , Layer LE , Kvello A , Reschke M , Spanagel R , Sprengel R *et al* (2003) Impaired long‐term memory and NR2A‐type NMDA receptor‐dependent synaptic plasticity in mice lacking c‐Fos in the CNS. J Neurosci 23, 9116–9122. 14534245 10.1523/JNEUROSCI.23-27-09116.2003 PMC6740829

[78] Tischmeyer W and Grimm R (1999) Activation of immediate early genes and memory formation. *CMLS* . Cell Mol Life Sci 55, 564–574. 10357227 10.1007/s000180050315 PMC11146814

[79] Wu H , Wang MC and Bohmann D (2009) JNK protects *Drosophila* from oxidative stress by trancriptionally activating autophagy. Mech Dev 126, 624–637. 19540338 10.1016/j.mod.2009.06.1082 PMC2750887

[80] Berke B , Wittnam J , McNeill E , Van Vactor DL and Keshishian H (2013) Retrograde BMP signaling at the synapse: a permissive signal for synapse maturation and activity‐dependent plasticity. J Neurosci 33, 17937–17950. 24198381 10.1523/JNEUROSCI.6075-11.2013 PMC3818560

[81] Oswald M , Brooks PS , Zwart MF and Mukherjee A (2016) Reactive oxygen species regulate activity‐dependent neuronal structural plasticity|bioRxiv. bioRxiv [Preprint]. 10.7554/eLife.39393 PMC6307858 30540251

[82] Hongpaisan J , Winters CA and Andrews SB (2003) Calcium‐dependent mitochondrial superoxide modulates nuclear CREB phosphorylation in hippocampal neurons. Mol Cell Neurosci 24, 1103–1115. 14697672 10.1016/j.mcn.2003.09.003

[83] Hongpaisan J , Winters CA and Andrews SB (2004) Strong calcium entry activates mitochondrial superoxide generation, upregulating kinase signaling in hippocampal neurons. J Neurosci 24, 10878–10887. 15574738 10.1523/JNEUROSCI.3278-04.2004 PMC6730216

[84] Zwart MF , Randlett O , Evers JF and Landgraf M (2013) Dendritic growth gated by a steroid hormone receptor underlies increases in activity in the developing *Drosophila* locomotor system. Proc Natl Acad Sci USA 110, E3878–E3887. 24043825 10.1073/pnas.1311711110 PMC3791713

[85] Meulener M , Whitworth AJ , Armstrong‐Gold CE , Rizzu P , Heutink P , Wes PD , Pallanck LJ and Bonini NM (2005) *Drosophila* DJ‐1 mutants are selectively sensitive to environmental toxins associated with Parkinson's disease. Curr Biol 15, 1572–1577. 16139213 10.1016/j.cub.2005.07.064

[86] Wilson MA (2011) The role of cysteine oxidation in DJ‐1 function and dysfunction. Antioxid Redox Signal 15, 111–122. 20812780 10.1089/ars.2010.3481 PMC3110098

[87] Jordán‐Álvarez S , Fouquet W , Sigrist SJ and Acebes A (2012) Presynaptic PI3K activity triggers the formation of glutamate receptors at neuromuscular terminals of *Drosophila* . J Cell Sci 125, 3621–3629. 22505608 10.1242/jcs.102806

[88] Cuesto G , Enriquez‐Barreto L , Caramés C , Cantarero M , Gasull X , Sandi C , Ferrús A , Acebes A and Morales M (2011) Phosphoinositide‐3‐kinase activation controls synaptogenesis and spinogenesis in hippocampal neurons. J Neurosci 31, 2721–2733. 21414895 10.1523/JNEUROSCI.4477-10.2011 PMC6623769

[89] Martín‐Peña A , Acebes A , Rodríguez J‐R , Sorribes A , de Polavieja GG , Fernández‐Fúnez P and Ferrús A (2006) Age‐independent synaptogenesis by phosphoinositide 3 kinase. J Neurosci 26, 10199–10208. 17021175 10.1523/JNEUROSCI.1223-06.2006 PMC6674615

[90] Kumar V , Zhang M‐X , Swank MW , Kunz J and Wu GY (2005) Regulation of dendritic morphogenesis by Ras‐PI3K‐Akt‐mTOR and Ras‐MAPK signaling pathways. J Neurosci 25, 11288–11299. 16339024 10.1523/JNEUROSCI.2284-05.2005 PMC6725910

[91] Meulener MC , Xu K , Thomson L , Thompson L , Ischiropoulos H and Bonini NM (2006) Mutational analysis of DJ‐1 in *Drosophila* implicates functional inactivation by oxidative damage and aging. Proc Natl Acad Sci USA 103, 12517–12522. 16894167 10.1073/pnas.0601891103 PMC1533799

[92] Bindokas VP , Jordán J , Lee CC and Miller RJ (1996) Superoxide production in rat hippocampal neurons: selective imaging with hydroethidine. J Neurosci 16, 1324–1336. 8778284 10.1523/JNEUROSCI.16-04-01324.1996 PMC6578569

[93] Dugan LL , Sensi SL , Canzoniero LM , Handran SD , Rothman SM , Lin TS , Goldberg MP and Choi DW (1995) Mitochondrial production of reactive oxygen species in cortical neurons following exposure to N‐methyl‐D‐aspartate. J Neurosci 15, 6377–6388. 7472402 10.1523/JNEUROSCI.15-10-06377.1995 PMC6578019

[94] Brennan AM , Won Suh S , Joon Won S , Narasimhan P , Kauppinen TM , Lee H , Edling Y , Chan PH and Swanson RA (2009) NADPH oxidase is the primary source of superoxide induced by NMDA receptor activation. Nat Neurosci 12, 857–863. 19503084 10.1038/nn.2334 PMC2746760

[95] Sorce S , Stocker R , Seredenina T , Holmdahl R , Aguzzi A , Chio A , Depaulis A , Heitz F , Olofsson P , Olsson T *et al* (2017) NADPH oxidases as drug targets and biomarkers in neurodegenerative diseases: what is the evidence? Free Radic Biol Med 112, 387–396. 28811143 10.1016/j.freeradbiomed.2017.08.006

[96] Bánfi B , Tirone F , Durussel I , Knisz J , Moskwa P , Molnár GZ , Krause K‐H and Cox JA (2004) Mechanism of Ca2+ activation of the NADPH oxidase 5 (NOX5). J Biol Chem 279, 18583–18591. 14982937 10.1074/jbc.M310268200

[97] Pandey D , Gratton J‐P , Rafikov R , Black SM and Fulton DJR (2011) Calcium/calmodulin‐dependent kinase II mediates the phosphorylation and activation of NADPH oxidase 5. Mol Pharmacol 80, 407–415. 21642394 10.1124/mol.110.070193 PMC3164331

[98] Tirone F and Cox JA (2007) NADPH oxidase 5 (NOX5) interacts with and is regulated by calmodulin. FEBS Lett 581, 1202–1208. 17346712 10.1016/j.febslet.2007.02.047

[99] Cabungcal J‐H , Steullet P , Morishita H , Kraftsik R , Cuenod M , Hensch TK and Do KQ (2013) Perineuronal nets protect fast‐spiking interneurons against oxidative stress. Proc Natl Acad Sci USA 110, 9130–9135. 23671099 10.1073/pnas.1300454110 PMC3670388

[100] Do KQ , Cuenod M and Hensch TK (2015) Targeting oxidative stress and aberrant critical period plasticity in the developmental trajectory to schizophrenia. Schizophr Bull 41, 835–846. 26032508 10.1093/schbul/sbv065 PMC4466197

[101] Beasley CL and Reynolds GP (1997) Parvalbumin‐immunoreactive neurons are reduced in the prefrontal cortex of schizophrenics. Schizophr Res 24, 349–355. 9134596 10.1016/s0920-9964(96)00122-3

[102] Hashemi E , Ariza J , Rogers H , Noctor SC and Martínez‐Cerdeño V (2017) The number of parvalbumin‐expressing interneurons is decreased in the medial prefrontal cortex in autism. Cereb Cortex 27, 1931–1943. 26922658 10.1093/cercor/bhw021 PMC6074948

[103] Lewis DA , Curley AA , Glausier JR and Volk DW (2012) Cortical parvalbumin interneurons and cognitive dysfunction in schizophrenia. Trends Neurosci 35, 57–67. 22154068 10.1016/j.tins.2011.10.004 PMC3253230

[104] Steullet P , Cabungcal J‐H , Coyle J , Didriksen M , Gill K , Grace AA , Hensch TK , LaMantia A‐S , Lindemann L , Maynard TM *et al* (2017) Oxidative stress‐driven parvalbumin interneuron impairment as a common mechanism in models of schizophrenia. Mol Psychiatry 22, 936–943. 28322275 10.1038/mp.2017.47 PMC5491690

[105] Bliss TV and Lømo T (1973) Long‐lasting potentiation of synaptic transmission in the dentate area of the anaesthetized rabbit following stimulation of the perforant path. J Physiol (Lond) 232, 331–356. 4727084 10.1113/jphysiol.1973.sp010273 PMC1350458

[106] Herring BE and Nicoll RA (2016) Long‐term potentiation: from CaMKII to AMPA receptor trafficking. Annu Rev Physiol 78, 351–365. 26863325 10.1146/annurev-physiol-021014-071753

[107] Klann E , Roberson ED , Knapp LT and Sweatt JD (1998) A role for superoxide in protein kinase C activation and induction of long‐term potentiation. J Biol Chem 273, 4516–4522. 9468506 10.1074/jbc.273.8.4516

[108] Klann E (1998) Cell‐permeable scavengers of superoxide prevent long‐term potentiation in hippocampal area CA1. J Neurophysiol 80, 452–457. 9658063 10.1152/jn.1998.80.1.452

[109] Thiels E , Urban NN , Gonzalez‐Burgos GR , Kanterewicz BI , Barrionuevo G , Chu CT , Oury TD and Klann E (2000) Impairment of long‐term potentiation and associative memory in mice that overexpress extracellular superoxide dismutase. J Neurosci 20, 7631–7639. 11027223 10.1523/JNEUROSCI.20-20-07631.2000 PMC6772863

[110] Gahtan E , Auerbach JM , Groner Y and Segal M (1998) Reversible impairment of long‐term potentiation in transgenic Cu/Zn‐SOD mice. Eur J Neurosci 10, 538–544. 9749716 10.1046/j.1460-9568.1998.00058.x

[111] Knapp LT and Klann E (2002) Potentiation of hippocampal synaptic transmission by superoxide requires the oxidative activation of protein kinase C. J Neurosci 22, 674–683. 11826097 10.1523/JNEUROSCI.22-03-00674.2002 PMC6758495

[112] Lee KY , Chung K and Chung JM (2010) Involvement of reactive oxygen species in long‐term potentiation in the spinal cord dorsal horn. J Neurophysiol 103, 382–391. 19906875 10.1152/jn.90906.2008 PMC2807226

[113] Fujii H and Hirano T (2002) Calcineurin regulates induction of late phase of cerebellar long‐term depression in rat cultured Purkinje neurons. Eur J Neurosci 16, 1777–1788. 12431231 10.1046/j.1460-9568.2002.02235.x

[114] Ferri A , Gabbianelli R , Casciati A , Paolucci E , Rotilio G and Carrì MT (2000) Calcineurin activity is regulated both by redox compounds and by mutant familial amyotrophic lateral sclerosis‐superoxide dismutase. J Neurochem 75, 606–613. 10899935 10.1046/j.1471-4159.2000.0750606.x

[115] Ferri A , Gabbianelli R , Casciati A , Celsi F , Rotilio G and Carrì MT (2001) Oxidative inactivation of calcineurin by Cu, Zn superoxide dismutase G93A, a mutant typical of familial amyotrophic lateral sclerosis. J Neurochem 79, 531–538. 11701756 10.1046/j.1471-4159.2001.00558.x

[116] Namgaladze D , Hofer HW and Ullrich V (2002) Redox control of calcineurin by targeting the binuclear Fe(2+)‐Zn(2+) center at the enzyme active site. J Biol Chem 277, 5962–5969. 11741966 10.1074/jbc.M111268200

[117] Wu KLH , Wu C‐A , Wu C‐W , Chan SHH , Chang AYW and Chan JYH (2013) Redox‐sensitive oxidation and phosphorylation of PTEN contribute to enhanced activation of PI3K/Akt signaling in rostral ventrolateral medulla and neurogenic hypertension in spontaneously hypertensive rats. Antioxid Redox Signal 18, 36–50. 22746319 10.1089/ars.2011.4457 PMC3503464

[118] Kim W , Youn H , Kang C and Youn B (2015) Inflammation‐induced radioresistance is mediated by ROS‐dependent inactivation of protein phosphatase 1 in non‐small cell lung cancer cells. Apoptosis 20, 1242–1252. 26033480 10.1007/s10495-015-1141-1

[119] O'Loghlen A , Pérez‐Morgado MI , Salinas M and Martín ME (2003) Reversible inhibition of the protein phosphatase 1 by hydrogen peroxide. Potential regulation of eIF2 alpha phosphorylation in differentiated PC12 cells. Arch Biochem Biophys 417, 194–202. 12941301 10.1016/s0003-9861(03)00368-0

[120] Crossthwaite AJ , Hasan S and Williams RJ (2002) Hydrogen peroxide‐mediated phosphorylation of ERK1/2, Akt/PKB and JNK in cortical neurones: dependence on Ca(2+) and PI3‐kinase. J Neurochem 80, 24–35. 11796740 10.1046/j.0022-3042.2001.00637.x

[121] Zhang L and Jope RS (1999) Oxidative stress differentially modulates phosphorylation of ERK, p38 and CREB induced by NGF or EGF in PC12 cells. Neurobiol Aging 20, 271–278. 10588574 10.1016/s0197-4580(99)00049-4

[122] Giniatullin AR and Giniatullin RA (2003) Dual action of hydrogen peroxide on synaptic transmission at the frog neuromuscular junction. J Physiol (Lond) 552, 283–293. 12897166 10.1113/jphysiol.2003.050690 PMC2343314

[123] Tsentsevitsky A , Nikolsky E , Giniatullin R and Bukharaeva E (2011) Opposite modulation of time course of quantal release in two parts of the same synapse by reactive oxygen species. Neuroscience 189, 93–99. 21627983 10.1016/j.neuroscience.2011.05.033

[124] Giniatullin AR , Darios F , Shakirzyanova A , Davletov B and Giniatullin R (2006) SNAP25 is a pre‐synaptic target for the depressant action of reactive oxygen species on transmitter release. J Neurochem 98, 1789–1797. 16945102 10.1111/j.1471-4159.2006.03997.x

[125] Orr BO , Fetter RD and Davis GW (2017) Retrograde semaphorin‐plexin signalling drives homeostatic synaptic plasticity. Nature 550, 109–113. 28953869 10.1038/nature24017 PMC5907800

[126] Khan SJ , Abidi SNF , Skinner A , Tian Y and Smith‐Bolton RK (2017) The *Drosophila* Duox maturation factor is a key component of a positive feedback loop that sustains regeneration signaling. PLoS Genet 13, e1006937. 28753614 10.1371/journal.pgen.1006937 PMC5550008

[127] Breckwoldt MO , Armoundas AA , Aon MA , Bendszus M , O'Rourke B , Schwarzländer M , Dick TP and Kurz FT (2016) Mitochondrial redox and pH signaling occurs in axonal and synaptic organelle clusters. Sci Rep 6, 23251. 27000952 10.1038/srep23251 PMC4802380

[128] Morgan B , Van Laer K , Owusu TNE , Ezeriņa D , Pastor‐Flores D , Amponsah PS , Tursch A and Dick TP (2016) Real‐time monitoring of basal H2O2 levels with peroxiredoxin‐based probes. Nat Chem Biol 12, 437–443. 27089028 10.1038/nchembio.2067

[129] Zhao Y , Hu Q , Cheng F , Su N , Wang A , Zou Y , Hu H , Chen X , Zhou H‐M , Huang X *et al* (2015) SoNar, a highly responsive NAD+/NADH sensor, allows high‐throughput metabolic screening of anti‐tumor agents. Cell Metab 21, 777–789. 25955212 10.1016/j.cmet.2015.04.009 PMC4427571

[130] Bilan DS , Pase L , Joosen L , Gorokhovatsky AY , Ermakova YG , Gadella TWJ , Grabher C , Schultz C , Lukyanov S and Belousov VV (2013) HyPer‐3: a genetically encoded H(2)O(2) probe with improved performance for ratiometric and fluorescence lifetime imaging. ACS Chem Biol 8, 535–542. 23256573 10.1021/cb300625g

[131] Kim CK , Adhikari A and Deisseroth K (2017) Integration of optogenetics with complementary methodologies in systems neuroscience. Nat Rev Neurosci 18, 222–235. 28303019 10.1038/nrn.2017.15 PMC5708544

[132] Bienert GP , Møller ALB , Kristiansen KA , Schulz A , Møller IM , Schjoerring JK and Jahn TP (2007) Specific aquaporins facilitate the diffusion of hydrogen peroxide across membranes. J Biol Chem 282, 1183–1192. 17105724 10.1074/jbc.M603761200

[133] Vieceli Dalla Sega F , Zambonin L , Fiorentini D , Rizzo B , Caliceti C , Landi L , Hrelia S and Prata C (2014) Specific aquaporins facilitate Nox‐produced hydrogen peroxide transport through plasma membrane in leukaemia cells. Biochim Biophys Acta 1843, 806–814. 24440277 10.1016/j.bbamcr.2014.01.011

